# Trial of pranlukast inhibitory effect for cedar exposure using an OHIO chamber

**DOI:** 10.3109/21556660.2012.703630

**Published:** 2012-06-17

**Authors:** Shuichiro Endo, Minoru Gotoh, Kimihiro Okubo, Kazuhiro Hashiguchi, Hidenori Suzuki, Keisuke Masuyama

**Affiliations:** 1 Otolaryngology, University of YamanashiJapan; 2 Department of Otorhinolaryngology, Nippon Medical School Chiba Hokusoh HospitalJapan; 3 Department of Otorhinolaryngology, Nippon Medical SchoolJapan; 4 Department of Otorhinolaryngology, Kitasato Institute HospitalJapan; 5 Pharmacodynamics, Nippon Medical SchoolJapan

**Keywords:** Cedar pollinosis, Crossover trial, Nasal symptoms, OHIO chamber, Pranlukast

## Abstract

**Objective:**

In practical guidelines for management of allergic rhinitis in Japan, pranlukast is a leukotriene receptor antagonist recommended for the treatment of pollinosis. However, the effect of pranlukast on nasal symptoms for cedar pollinosis has not been thoroughly investigated. The aim of this study is to examine this effect in a double-blind controlled crossover study using a pollen challenge chamber (the OHIO Chamber) developed in Japan.

**Research design and methods:**

A total of 39 patients with cedar pollinosis were targeted. The subjects were exposed to a specific amount of cedar pollen (8000/m^3^) in the OHIO Chamber during the non-cedar pollen season. Efficacy of pranlukast for the treatment of artificially induced nasal symptoms was compared with that of a placebo using the crossover method. Pranlukast was administered orally for 3 days, after dinner on the day before cedar pollen exposure, after breakfast and after dinner on the day of cedar pollen exposure, and after breakfast on the following day. Pollen testing was carried out twice, with a 1-week wash-out interval.

**Clinical trial registration:**

The University Hospital Medical Information Network in Japan (UMIN), number UMIN000001282.

**Main outcome measures:**

The effect of pranlukast was evaluated using self-rating of nasal symptoms by the subjects.

**Results:**

All 39 subjects demonstrated a positive skin reaction to cedar pollen by a positive CAP-RAST score (class 2 or higher) within the last 3 years, and experienced aggravated congestion during the cedar pollen season for more than 2 years. Nasal congestion was inhibited significantly in the pranlukast group compared to the placebo group during cedar pollen exposure. Furthermore, pranlukast significantly inhibited nasal congestion compared to the placebo on the day after exposure and on the following day.

**Conclusions:**

The effect of pranlukast on cedar pollinosis indicates immediate action, and such an effect could take place continuously after cedar pollen exposure. These results demonstrate that pranlukast is effective for the relief of congestion due to pollinosis.

## Introduction

Pollinosis in Japan is the condition in which plant pollen, from sources such as Japanese cedar and Japanese cypress, induces allergic symptoms such as sneezing, runny nose, congestion, and itchy eyes. This disease is also called seasonal allergic rhinitis. Due to the recent worldwide increase in the number of patients with pollinosis, this condition has become a current issue^1^. In Japan, the increase in the number of patients with cedar pollinosis has become a social problem. During the pollen season, the airborne cedar pollen concentration is high and has a wide dispersion, resulting in a high nationwide prevalence of cedar pollinosis and marked discomfort in patients^2,3^. Cedar pollinosis occurs annually from February to April in Japan. The prevalence of nasal allergies has increased each year and has reached 39.6% according to epidemiological surveys carried out in 2006 and 2007^3^. Nasal allergy greatly affects the daily activities of the sufferer and the development of an effective treatment is needed.

Pranlukast is the first CysLT_1_ (cysteinyl leukotriene) receptor antagonist discovered in Japan^4^ and the first to be introduced as a clinical leukotriene receptor antagonist. Efficacy of pranlukast for adult bronchial asthma^5^, pediatric bronchial asthma^6^, and allergic rhinitis^7^ has been demonstrated. In the otorhinolaryngological field, Okuda *et al*. was able to demonstrate the usefulness of pranlukast in the treatment of perennial allergic rhinitis in a double-blind controlled study using epinastine hydrochloride as a control. Efficacy of pranlukast for nasal congestion has especially gained attention^8^. The clinical effect of pranlukast on cedar pollinosis has been demonstrated^9,10^, but not fully investigated, despite the prevalence of pollinosis in Japan.

Treatment of pollinosis with antihistamines is generally recommended according to international standard guidelines such as ARIA^11^, but these guidelines differ slightly from those in Japan^12^. According to international guidelines, pollinosis is classified only by severity, whereas in the Japanese guideline, the importance of symptom type, in addition to severity, is emphasized for drug selection. Consequently pollinosis with aggravated congestion type is often treated with anti-leukotrienes in combination with other drugs in Japan. However, evidence regarding the efficacy of anti-leukotrienes without other drugs for congestion due to pollinosis has not been demonstrated. There are also a few reports which show that the effect of anti-leukotrienes in a challenge chamber is weaker than that of antihistamines^13^. Under such a background, we decided to investigate the effect of pranlukast without other drugs on pollinosis symptoms induced by cedar pollen exposure in an OHIO Chamber^14^, a standardized pollen challenge chamber, as compared with a placebo.

## Patients and methods

### Subjects

A total of 39 adult patients with congestion-type or combined-type cedar pollinosis were targeted. Inclusion criteria were ages between 20 and 65 years, presence of symptoms during the cedar pollen season for 2 years or more, and positive for antibodies against cedar pollen class 2 or higher but a negative or class 1 response against house dust and mites by antigen-specific serum IgE antibody test (capsulated hydrophilic carrier polymer radioallergosorbent test [CAP-RAST]) within the last 3 years. Exclusion criteria were the presence of lesions in the nasal or eye mucosa, a steroid injection within the past 6 months, the presence of nasal disease (septonasal deviation, nasal polyp, etc.), systemic disease (asthma, tuberculosis, etc.), and a past medical history of anaphylaxis or hypersensitivity to ingredients in pranlukast or the placebo capsules. Pregnant and breast-feeding women, as well as women seeking pregnancy during the study period, and patients judged ineligible for participation in the study by the clinical investigators, were also excluded. The study was performed in conformance with the Declaration of Helsinki and the Ethics Guidelines Concerning Clinical Studies. The study protocol was examined and approved by the Institutional Review Board of Samoncho Clinic and written consent was obtained from all subjects. This study is registered with the University Hospital Medical Information Network in Japan (UMIN), number UMIN000001282.

### Study schedule

**Figure 1.**
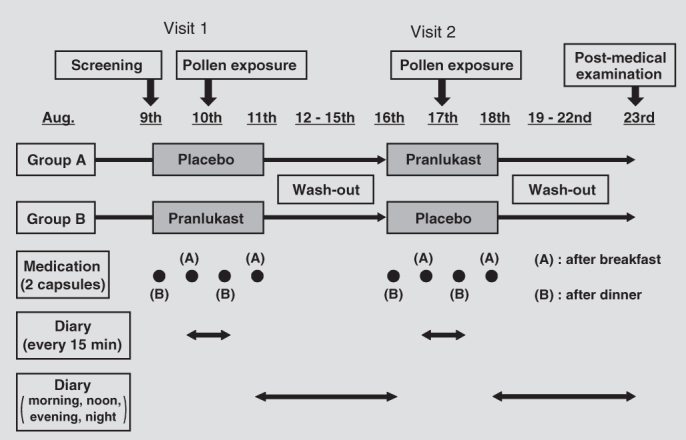
Study schedule. The study periods consisted of visit 1, washout and visit 2. Eligible subjects were enrolled in the treatment period and randomized to receive pranlukast or placebo for 3 days (visit 1) followed by a washout period of 7 days, and then crossed over to the other treatment period for 3 days (visit 2). Subjects were exposed to cedar pollen in the OHIO chamber for 3 hours on the second day of each treatment period.

This study was performed as a randomized, double-blind, placebo-controlled crossover trial in August, during the non-cedar pollen season. Subjects were randomly divided into two groups – one group which received two pranlukast capsules containing 112.5 mg/capsule and one group which received two placebo capsules. Pranlukast or the placebo was administered orally for 3 days, after dinner on the day before cedar pollen exposure, after breakfast and after dinner on the day of cedar pollen exposure, and after breakfast on the following (Figure 1). Cedar pollen concentration in the OHIO Chamber was set at 8000/m^3^. Pollen was counted by laser particle counter (KC-20) and the concentration within the chamber was computer-controlled. Temperature and humidity in the chamber were 22°C and 45%, respectively. This chamber can accommodate a maximum of 13 persons. Subjects were exposed to cedar pollen for 3 hours, but could exit the chamber if symptoms worsened during exposure.

### Evaluation items

Primary endpoint: Nasal symptoms (sneezing, runny nose, congestion, and/or nasal itching) and eye symptoms (itchy and/or watery eyes) were each self-rated by the subjects every 15 minutes based on the following scale: 0, none; 1, mild; 2, moderate; 3, severe; 4, most severe. The total score for sneezing, runny nose, and congestion was determined to be the nasal symptoms score.

### Statistical analysis

*T*-test and log-rank test were used, with *p* < 0.05 as the significance level.

## Results

### Patient background (Table 1)

In all, 39 patients with cedar pollinosis who met the inclusion criteria and who were not in conflict with the exclusion criteria participated in the study. Patients included 14 males and 25 females, 20–58 years old, with a mean age of 35.8 years. A total of 20 patients received pranlukast during the first half and a placebo during the second half of the test, and 19 patients received a placebo in the first half and pranlukast in the second half of the cross-over study (Figure 1). In the following sections, results for the pranlukast group refer to the combined data for patients receiving pranlukast in both parts of the trial, and results for the placebo group similarly refer to the combined data in both parts of the trial.

**Table 1.** Patient background.

Number of patients	39		
Gender			
Male	14		35.9%
Female	25		64.1%
Age (years)			
Min–max		20–58	
Mean ± SD		35.8 ± 9.7	
CAP-RAST			
Class 2	6		15.4%
Class 3	18		46.2%
Class 4	9		23.1%
Class 5	6		15.4%
Class 6	0		0%

CAP-RAST: capsulated hydrophilic carrier polymer radioallergosorbent test.

### Nasal symptom scores

**Figure 2.**
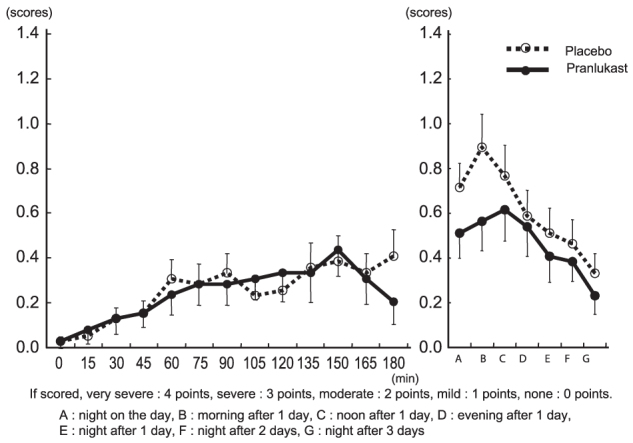
Changes in sneezing score.

Sneezing: During exposure in the pollen challenge chamber, the sneezing score was ≤0.5 in both the pranlukast and placebo groups, indicating mild symptoms and no difference between groups. On the following morning, the score increased in the placebo group, but was inhibited slightly in the pranlukast group (0.897 vs. 0.564, *p* = 0.094) (Figure 2).

**Figure 3.**
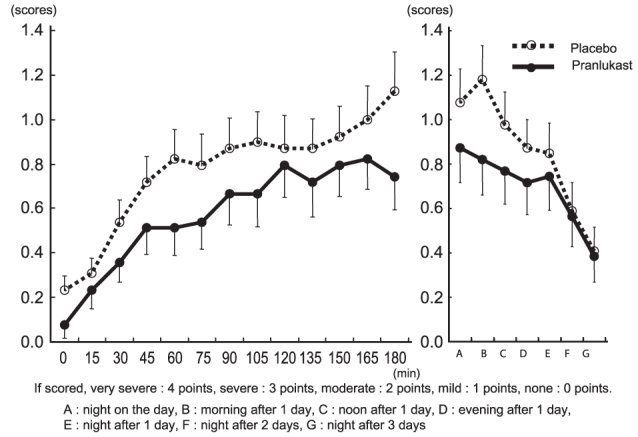
Changes in nasal discharge score.

Nasal discharge: During exposure in the pollen challenge chamber, the nasal discharge score increased over time and was ≥1 after 165 minutes in the placebo group. In the pranlukast group, the score was one scale below that of the placebo group. After subjects left the chamber, the score increased continuously until the following morning in the placebo group, but tended to decrease in the pranlukast group (1.179 vs. 0.821, *p* = 0.106) (Figure 3).

**Figure 4.**
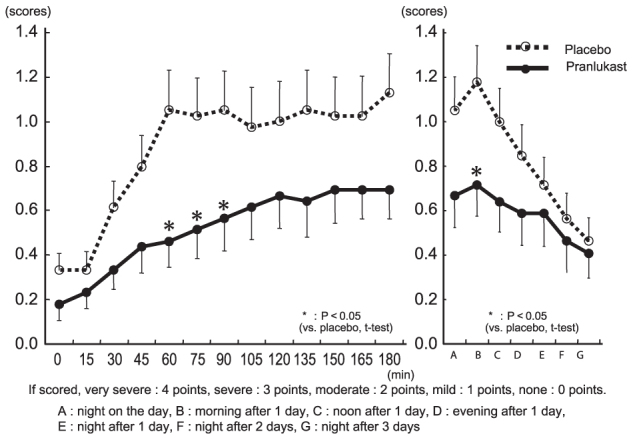
Changes in nasal congestion score.

Congestion: During exposure in the pollen challenge chamber, the congestion score increased over time and reached 1 after 60 minutes in the placebo group. In the pranlukast group, this increase was inhibited and the score was significantly lower than that in the placebo group at 60 minutes (1.051 vs. 0.462, *p* = 0.008), 75 minutes (1.026 vs. 0.513, *p* = 0.020), 90 minutes (1.051 vs. 0.564, *p* = 0.036), and 180 minutes (1.128 vs. 0.692, *p* = 0.053). After the subjects left the chamber, the score increased continuously until the following morning in the placebo group, but was significantly inhibited in the pranlukast group (1.179 vs. 0.718, *p* = 0.037) (Figure 4).

Nasal itching: During exposure in the pollen challenge chamber, the nasal itching score was <0.8 in both groups, indicating mild symptoms and no significant difference between groups. After leaving the chamber, the score increased at night in the placebo group, but tended to decrease in the pranlukast group and became significantly lower than that of the placebo group at night on the day of exposure (0.667 vs. 0.333, *p* = 0.025).

**Figure 5.**
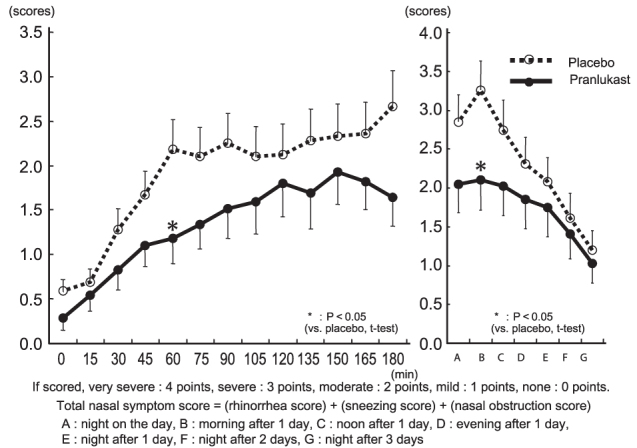
Changes in total nasal symptom score.

Total nasal symptom score: During exposure in the pollen challenge chamber, the score increased over time and exceeded 2.5 at 180 minutes in the placebo group. In the pranlukast group, this increase was inhibited and the score was significantly lower than that of the placebo group at 60 minutes (2.179 vs. 1.184, *p* = 0.030), and tended to be lower at 180 minutes (2.667 vs. 1.641, *p* = 0.050). After the subjects left the chamber, the score increased continuously until the following morning in the placebo group, but this increase was significantly inhibited on the following morning in the pranlukast group (3.256 vs. 2.103, *p* = 0.038) (Figure 5).

### Total eye symptoms scores

Itchy eyes: During exposure in the pollen challenge chamber, score for itchy eyes increased slightly to about 0.5 and 0.3 in the placebo and pranlukast groups, respectively, with no significant difference between the groups. After the subjects left the chamber, the scores decreased in both groups. The score was significantly lower on the following afternoon in the pranlukast compared to the placebo group (0.154 vs. 0.436, *p* = 0.039).

Watery eyes: During and after exposure in the pollen challenge chamber, score for water eyes was ≤ 0.2 in both groups, indicating that this symptom was very mild or absent.

### Cough symptom score

During and after exposure in the pollen challenge chamber, the score for coughing was about 0.2 in both groups, indicating that this symptom was very mild or absent.

## Discussion

This study was performed using the OHIO Chamber for artificial dispersal of pollen. This chamber can accommodate 13 persons and provides consistent cedar pollen exposure, so changes in subjective symptoms and objective data can be investigated^14^. Our results showed that development of nasal symptoms was significantly inhibited by pranlukast compared to the placebo. In particular, congestion during pollen exposure was markedly inhibited by pranlukast. Antihistamines are generally thought to be effective for histamine-induced sneezing and runny nose, but no apparent effect on congestion has been reported. Pranlukast significantly improved congestion in a double-blind study performed on perennial allergic rhinitis patients, using epinastine hydrochloride as a control, and in a placebo-controlled clinical pharmacology study with antigen sensitization^8^. The effect of pranlukast on congestion in pollinosis as shown in this study, suggests that pranlukast inhibits LTs produced by mast cells or eosinophils in the immediate phase, and LTs produced by eosinophils in the late phase. The effect on sneezing and runny nose in the late phase may be the result of improved nasal mucosal hypersensitivity through the inhibition of LT action by migrating cells, especially eosinophils.

Efficacy of pranlukast on allergic rhinitis appears 1–2 weeks after oral administration^7,8^. In this study, patients treated with pranlukast after dinner on the day before cedar pollen exposure and after breakfast on the day of exposure had reduced congestion and lower nasal symptom scores during pollen exposure. Furthermore, continuous treatment with pranlukast on the night after exposure and on the following morning significantly inhibited nasal symptoms, including congestion at night on the exposure day until noon on the following day. This suggests that treatment with pranlukast before cedar pollen season may inhibit symptoms, in other words demonstrate efficacy by preventive administration. These results also suggest the need for continuous medication.

Patients with cedar pollinosis were treated with pranlukast after dinner on the day before cedar pollen exposure in the OHIO Chamber and after breakfast on the day of exposure. As a result, cedar pollen-induced development of symptoms could be inhibited. In addition, continuous treatment after dinner following exposure and after breakfast on the following morning could inhibit recurrence of nasal and eye symptoms after cedar pollen exposure. In a pollen exposure study using a different anti-leukotriene agent, montelukast, efficacy could be noted but at an inferior level to that of antihistamines^13^. Patients with pollinosis and congestion symptoms were selected according to PG-MARJ recommendations, and the effect of pranlukast on congestion and inhibition of nonspecific hypersensitivity in the late phase could be demonstrated. Thus, in addition to the severity, diagnosis of symptom type is considered important in the selection of the appropriate drug for pollinosis. A similar effect between pranlukast and montelukast could also be seen^15^, suggesting that the difference in results between the two challenge chamber studies could have been due to methodological differences.

## Conclusion

Anti-leukotrienes exhibit a marked therapeutic effect on congestion. These drugs are safe and cause no adverse events, whereas antihistamines may cause central sedation. Anti-leukotrienes also improve allergic inflammation caused by eosinophil release^16^, airway secretion^17^, and lower airway remodeling^18^. In this study, pranlukast showed a rapid effect on cedar pollinosis, especially for congestion, and continuous treatment was also effective for nasal symptoms including congestion. It is unclear whether the fast action of pranlukast is superior to antihistamines or not, and this may depend on the type of nasal symptoms. However, results at this time show that pranlukast is a useful drug for congestion in pollinosis.

## Transparency

### Declaration of financial/other relationships

There are no relationships to be declared for any of the authors.

The peer reviewers on this manuscript have disclosed that they have no relevant financial relationships.
